# Community-Based Intervention for Active Detection and Provision of Single-Dose Rifampicin Post-Exposure Prophylaxis to Household Contacts of Leprosy in Bolivia

**DOI:** 10.3390/tropicalmed9050101

**Published:** 2024-05-01

**Authors:** Abundio Baptista Mora, Nimer Ortuño-Gutiérrez, Deisy Zurita Paniagua, Carlos Hurtado Solares, Anil Fastenau, Christa Kasang

**Affiliations:** 1 German Leprosy and Tuberculosis Relief Association (GLRA), Sucre 0000, Bolivia; abu_bap@hotmail.com; 2 Damien Foundation, 1081 Brussels, Belgium; 3 Dermatological Hospital of Jorochito, Santa Cruz 00591, Bolivia; mayita170876@gmail.com; 4 Epidemiology, Departmental Health Service, Santa Cruz 00591, Bolivia; drcarloshurtados@hotmail.com; 5 German Leprosy and Tuberculosis Relief Association (GLRA), 97080 Wurtzbourg, Germany; anil.fastenau@dahw.de (A.F.); dr.christa.kasang@dahw.de (C.K.)

**Keywords:** single-dose rifampicin, post-exposure prophylaxis, low endemic, leprosy elimination, health knowledge, attitudes, practice, health education, community participation

## Abstract

Background: To achieve zero leprosy cases in Santa Cruz, Bolivia, we designed a community-based active detection and provision of single-dose rifampicin post-exposure prophylaxis (SDR-PEP) to household contacts with new leprosy patients. Methods: From July to August 2021, we assessed the current knowledge, attitude, and practices through structured interviews and focus group discussions with community representatives and health staff. This was followed by sensitization sessions, the training of health staff, and the reinforcement of referral mechanisms. Teams, including health staff and community volunteers, visited all new leprosy patients detected in 2021–2023 and household contacts. Results: Among 115 community representatives, knowledge about leprosy etiology was attributed to non-biological factors (74%); fear accounted for 77%, and access to care was perceived as weak (74%), but the outlook was improved by SDR-PEP (80%). Among the 217 health staff interviewed, the programmatic barriers identified were a lack of referral feedback (67%), limited supplies for diagnosis and prevention, and ineffective training (64%). We visited 70 new patients and 258 household contacts. The median age in household contacts was 25 years old; 49% were women, 98% were eligible for SDR-PEP, and all who were eligible accepted it. Those who were non-eligible included one tuberculosis patient and six newly detected leprosy patients (23‰). Conclusions: A community-based intervention was successful in Santa Cruz, Bolivia. Misbeliefs and a lack of knowledge were identified as barriers. Programmatic components should be reinforced for SDR-PEP extension.

## 1. Introduction

Leprosy is an ancient infectious disease affecting 200,000 new patients annually worldwide. Although leprosy is curable, delayed diagnoses and poor management of reactions cause permanent disability. It is estimated that four million persons affected by leprosy live with such disabilities. The Global Leprosy Strategy 2021–2030 aims to stop transmission, targeting 120 countries in which the goal of zero cases of autochthonous leprosy is to be achieved by 2030 [1]. In 2018, the World Health Organization (WHO) recommended single-dose rifampicin as post-exposure prophylaxis (SDR-PEP) to contacts of leprosy index cases without signs of leprosy [2,3]. This strategy contributes to curbing transmission and preventing the onset of disease and disability as it prevents infected persons from developing the disease. The prevention and active detection of leprosy in low-prevalence settings are important to achieve zero cases of autochthonous leprosy. In low-prevalence settings for leprosy, SDR-PEP has been effective in stopping transmission [4,5].

Bolivia, with a population of 12,224,000 million, is a low-endemic setting for leprosy. The number of new leprosy cases was reduced from 104 in 2012 to 38 in 2022, equivalent to a new cases notification rate per million of 10 and 1.7, respectively [6]. Although Bolivia seems to be close to the elimination of leprosy, children ≤14 years old are still detected among new cases (around 3% of new cases). Santa Cruz records more than 60% of new nationwide leprosy cases, and it borders Brazil, which is the most leprosy-prevalent country in South America [7]. The involvement of the Bolivian community in designing inclusive developments for people affected by disabilities (including leprosy) was the lowest in a study that reviewed community participation, which also studied Brazil and Colombia [8]. The Regional Health Secretary of Santa Cruz partnered with international non-governmental organizations, such as the German Leprosy and Tuberculosis Association (GLRA) and Damien Foundation Belgium, and community representatives for the integration of SDR-PEP in the control of leprosy. Operational research in designing the strategy that involves community representatives is needed to increase the acceptance and sustainability of the interventions.

In this study, we aimed to evaluate the feasibility of the provision of SDR-PEP to the household contacts of new leprosy patients in Santa Cruz while engaging representatives of the community to enhance acceptability. Specifically, we present the results of structured interviews and knowledge, attitudes, and practices of community representatives, including health staff, employed in the design of health messages to enhance acceptability. We also describe the number of persons present for screening for leprosy, the number and proportion who accepted physical examination, the number and proportion of cases wherein leprosy was detected, the number and proportion of those who were eligible for SDR-PEP, and the number and proportion of those who accepted SDR-PEP. The results of this study will guide the scaling up of the SDR-PEP intervention in the rest of the country, incorporating the lessons learned into a national Leprosy Elimination Roadmap in Bolivia.

## 2. Materials and Methods

### 2.1. Study Design

**Figure 1:**
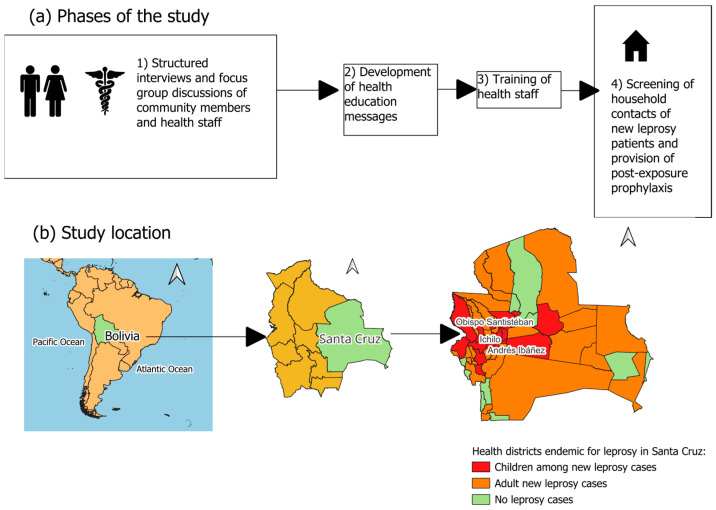
Implementation phases for active case detection of household contacts of new leprosy patients and provision of post-exposure prophylaxis in Santa Cruz, Bolivia, 2021–2023: (**a**) Phases of the study and (**b**) Study location.

This is a mixed-methods study that combines qualitative and quantitative approaches. To assess knowledge, attitudes, and practices and develop health education messages, we conducted structured interviews and focus group discussions (FGD) with representatives of the community and health staff. To assess active case detection and the feasibility of SDR-PEP, a cohort of new leprosy cases and their household contacts were recruited from 2021 to 2023 in three high-endemic districts in Santa Cruz, Bolivia (Figure 1).

### 2.2. Study Setting

Bolivia is a landlocked South American country that is categorized as within the medium human development category and ranked 120 among 193 countries by the Human Development Index (HDI) [9]. Bolivia is still struggling with socioeconomic and health challenges, such as poverty, economic and gender inequalities, exclusion and discrimination, drought and massive fires, and health crises such as the following epidemics: COVID-19 [10], yellow fever [11], and dengue [12].

#### Study Sites

This study was conducted in Santa Cruz, the largest of nine regions of Bolivia, with 370,621 km2 and a total estimated population of 3,486,624 in 2023 [13]. From 2013 to 2022, among 313 new leprosy cases reported in 56 municipalities, there were 62 (20%) children ≤14 years old in 16 municipalities. Whenever a new leprosy case is found, it is reported to one of the 16 health districts (“Coordinación Red de Salud = CRS”). The CRS network has 27 health facilities in primary care, and the referral is a Dermatological Hospital of Jorochito which serves as a national referral for dermatological diseases. Most of the leprosy cases are diagnosed passively at the Dermatological Hospital of Jorochito. Household screening for leprosy among household contacts is organized by an outreach medical team, but no provision of SDR-PEP was in place before the study initiation.

We included three CRSs endemic for leprosy (two rural Obispo Santisteban, Ichilo, and one urban East CRS from Andrés Ibañez), accounting for a total population of 848,840 inhabitants, that reported 183 cases, of which 42 (23%) were children ≤ 14 years from 2013 to 2022.

### 2.3. Participants

We enrolled index cases, meaning all new leprosy patients reported from 1 January 2021 to 31 December 2023 and their household contacts from three CRSs. Index cases and household contacts (all ages) were enrolled during door-to-door screening for leprosy from 29 September 2021 to 31 December 2023.

For the qualitative methods, we included the consent of community representatives aged ≥18 years old from three CRS endemic for leprosy recruited from 15 to 30 July 2021 for the structured interviews and from 30 July to 30 August 2021 for FGD. Health staff working in the health facilities of the CRSs interviewed were selected, with working experience of at least ≥3 years for medical doctors and ≥2 years for nurses.

After completing the qualitative part of the study and incorporating key messages, we conducted sensitization sessions for community representatives in the districts included in the study. We also conducted training of health staff from the most endemic municipalities and organized the referral to Jorochito Dermatological Hospital, ensuring that material for diagnosis was available at the health facilities of the districts included.

### 2.4. Sample Size

For the provision of SDR-PEP, we included all consent household members of new leprosy cases notified from 1 January 2021 to 31 December 2023 in three CRS.

For the qualitative study, a total of 230 potential participants were listed, and a convenience sampling of 115 participants was selected randomly for the structured interviews. For the FGD, we randomly selected at least 50% of those included in the structured interviews among those who have contact with a leprosy case in their household or neighborhood.

### 2.5. Data Collection

For the qualitative study, we conducted structured interviews that lasted around 20 min. We used two specific questionnaires developed by the team of investigators. One was for community representatives and included knowledge about leprosy disease and attitudes and practices regarding leprosy. The second questionnaire for health staff included the same questions for knowledge, attitude, and practice items including additional questions regarding management of leprosy patients according to the NLP guidelines.

For the FGD, five sessions were organized, including an average of 11 participants and lasting ≥ 60 min. Sessions were recorded. A trained moderator shared the topics according to the importance of topics generated by the structured interviews: knowledge about leprosy, access to healthcare, and community participation in prevention and disease control.

For the provision of SDR-PEP, we listed all leprosy cases treated according to the National Leprosy Program (NLP) guidelines from the leprosy register of CRS [14]. Two teams composed of a medical doctor and nurse experts on the clinical management of leprosy, health staff from the CRS, and community representatives visited new leprosy cases and household members at home. Sociodemographic data, clinical signs, provision of SDR-PEP, and household coordinates were collected using Open Data Kit Collect (ODK) during household visits of leprosy new cases and their household contacts. We cross-checked data entered into ODK with data from a paper-based form, including key variables such as eligibility for SDR-PEP according to WHO guidelines [3].

### 2.6. Data Analysis

Data about SDR-PEP were analyzed using STATA software (Version 15, StataCorp, College Station, TX, USA). Data from the structured interviews were entered into a customized database in Microsoft Excel version 2312, frequency of distribution of sociodemographics and themes was analyzed using the same software. In FGD, participants were distributed in small groups discussing specific topics and sharing free verbal opinions. Then, in the plenary, the moderator (Principal Investigator) developed a summary on a flipchart, blackboard, or a projector using tools such as Venn diagram and Likert scale. Finally, the results of the discussion were validated by the participants. The key findings were summarized in subthemes in the categories of knowledge, attitudes, and practices. Finally, health messages were identified and endorsed, and those were included in training sessions of health staff; their application was supervised during the visits to the households of leprosy patients.

## 3. Results

### 3.1. Structured Interviews

We interviewed 115 participants: 59% were female, 45% were 41–60 years old, 62% lived in rural areas, 75% spoke Spanish, and 39% had primary school education. Details are shown in Table 1.

Regarding health staff, we enrolled 217 for structured interviews, of which 142 (65%) were medical doctors and 75 were nurses. Among medical doctors, 68% were males, 34% belonged to the age category of 36–45 years old, and 46% had work experience of 4–6 years. Among nurses, 90% were female, 30% belonged to the age category of 36–45 years old, and 33% had a work experience of 4–6 years. The details of the sociodemographic characteristics of the health staff interviewed are displayed in Table 2.

#### 3.1.1. Knowledge

Among key informants, 45% acquired knowledge from a family or neighbor affected by leprosy. Clinical manifestations were unknown in 58%. Among those who expressed knowledge of infectivity and mutilation, the following was mentioned:

*“(…) I have only heard, they say it is serious and very contagious (…) people’s flesh falls off; they become blind and rot to death (…)”*

Key informants attributed the etiology to superstition, witchcraft, spells, and other non-biological factors (74%). Health staff were aware of the etiology and clinical manifestations.

Health staff’s main source of knowledge about leprosy was working experience (51%). However, experienced doctors expressed deficiencies in the knowledge of new staff:

*“For novices leprosy is associated with large lesions and disabilities, they do not realize that its onset is slow and confused with any fungal or bacterial lesion; they do not know that the loss of sensitivity is key in the diagnosis”*

Also, health staff lack the knowledge for conducting contact screening:

*“(…) We know that leprosy exists in our territory, but we do not know where they live to visit them.”*

#### 3.1.2. Attitude

Fear was clearly expressed among key informants (77%) and linked to isolation to stop the spread of leprosy:

*“It’s a very contagious and deadly disease, that’s why they have him isolated, also his plate and his food have to be separated.”*

Most of the health staff do not manifest fear of leprosy. However, fear is still present among some of them:

*“I was trained when I worked in endemic areas of leprosy, so I know that it is a skin disease that disfigures and causes a lot of damage to the body. The relatives of a patient who lived years ago, who already died, told me that when he went to the health center, the doctors and nurses tried to avoid him, they thought it was highly contagious (…), and it is not like that, I explained to health staff that it is a disease like the others and that it has free treatment; but, I could not convince them”*. Medical doctor.

#### 3.1.3. Practices

Among key informants, isolation and hiding patients were strongly suggested, access to care was strongly perceived as poor (74%), and prevention through post-exposure prophylaxis by qualified health staff was strongly perceived as promising method to stop the disease (80%).

Health staff strongly denied fear of leprosy, acknowledged limited capacity for the management of leprosy, and perceived poor access to programmatic support (67% including supplies for diagnosis and care and feedback of referral system):

*“When I worked in an endemic zone I saw a case of leprosy that had only a few spots on the forearms and one on the face. The doctor said that it was a not very advanced case and that is why he did not present other larger lesions as we see in the photos; he was told to go to Jorochito Dermatological Hospital”*. Nurse.

*“When one of these leprosy cases occurs, we refer him to Santa Cruz, to CENETROP (National Center for Tropical Diseases), or Jorochito Dermatological Hospital. There is little we can do, besides the personnel would become susceptible”*. Medical doctor.

*“The leprosy patients who are treated in Jorochito Dermatological Hospital return to their community, but we don’t know anything because we don’t receive a referral form. We don’t know what treatment they follow or how they are monitored”*. Nurse.

Health staff perceived programmatic activities of prevention as poor (64%, including the provision of health education materials and supervision):

*“During supervisions by national health programs, leprosy is not discussed; only those who come from Epidemiology remind us that we must report leprosy because it is part of the mandatory epidemiological surveillance”*. Medical doctor.

We summarized the details of the knowledge, attitude, and practices of key informants and health staff in Table 3.

### 3.2. Focus Group Discussion (FGD)

Among 65 key informants that participated in FGD, 60% were female, 61% belonged to the age category of 26–40 years, 65% attended primary school, and 29% worked in the agriculture sector. We display in Table 4 the details of sociodemographic characteristics of key informants who participated in FGD.

#### 3.2.1. Knowledge

The FGD confirmed misconceptions and a lack of knowledge about the disease; some examples include the following:

*“I confused leprosy with some skin patches due to humidity, like any other that occurs in these warm areas.”*

*“I come from the Chaco, my parents know leprosy with another name “Pujyu”, it is the same that here they say it is leprosy”*

*“(…) leprosy has happened to him because he has been having children everywhere, he has been cursed or bewitched, that is why he has not been able to cure himself until he has died alone, without anyone to take care of him”.*

*“(…) my mother used to tell me that it was “cancha” because the dog or cat licks you and those skin lesions appear”.*

*“my neighbor says that he had leprosy because he ate snake meat in the bush (…)”.*

#### 3.2.2. Attitude

Some leprosy patients hide their disease:

*“My nephew had many spots on his body, some were big; in so much heat he wore a long sleeve shirt so we could not see them (…) they told me he was stung by “cepe”, which is a big black ant when he had fallen asleep near that nest”.*

Self-admittance to traditional healers is also one stop before reaching the health system:

*“When he fell sick their sons took him to be seen by a traditional healer (jampiri). As he has not improved (…), someone told him to go to Jorochito Dermatological Hospital, he is been treated there…”.*

#### 3.2.3. Practices

Management capacity in the health system is perceived as weak:

*“The doctors have studied and know about the diseases, but they do not know about others that are more dangerous (…) they have visited several doctors, they have traveled as far as Santa Cruz, (…) they have given him all kinds of medicines for more than 1 year, they have not been able to cure him (…)”.*

*“when she went to the doctor they only gave her pills and ointments, and she did not get better from those spots on her back and arms; the traditional healers have not been able to do anything either (…)”.*

The following concerns prevention and spread of health messages:

*“The media, do not teach about leprosy, there are messages about AIDS, Dengue, Covid, they even talk about distributing condoms, but leprosy is not heard”.*

*“There is little material that we receive about leprosy which is forgotten but is on the most serious disease. We have enough health material from other programs”.*

### 3.3. Screening of Leprosy and Uptake of SDR-PEP

**Figure 2:**
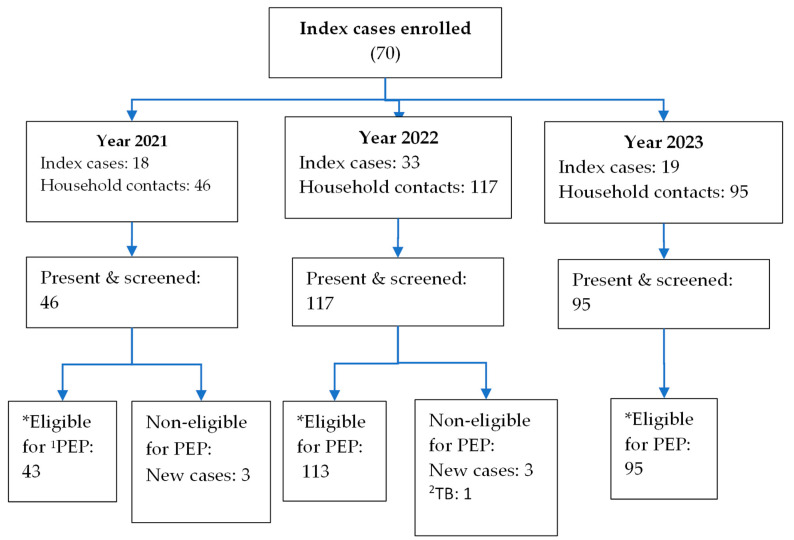
Flow chart of household members screened for leprosy; Santa Cruz, Bolivia, 2021–2023. ^1^PEP = Post-exposure prophylaxis. ^2^TB = Tuberculosis. * All eligible for PEP accepted it.

In the period of 29 September 2021 and 31 December 2023, we enrolled 70 index cases and 258 household contacts. Household contacts had a median of 25 years old with an interquartile range of 12–38, and 127 (49%) were women. All household members agreed to be screened for leprosy. Among 258 household contacts, 251 (97%) were positive for leprosy and accepted the provision of SDR-PEP. Non-eligible household contacts corresponded to six new leprosy cases and one TB patient under treatment. One eligible household contact receiving SDR-PEP reported minor adverse events. We found six new cases; all were women, one was 18 years old, five were between 30 and 49 years old, four had MB form, two had PB form, three had grade two disability, and the remaining three had no disability. The details of enrolment and screening are disclosed in Figure 2.

## 4. Discussion

In this pilot study, including three health districts, the yield of screening for leprosy was high, with a rate of 23 per 1000 (95% Confidence Interval 8–49). In low-endemic settings, systematic household screening of contacts by qualified health staff is proven effective [15] and should be repeated until the elimination of the disease documented by the absence of autochthonous new leprosy patients, as recommended by the Leprosy Elimination Monitoring Tool [16]. However, as leprosy is also spread among neighbors, targeting areas for active detection where clustering is documented will be necessary in addition to systematic household contact screening and the provision of SDR-PEP [17]. Therefore, designing the roadmap for leprosy elimination in Bolivia should integrate mapping into the surveillance system.

To our knowledge, this is the first study in Bolivia that documented the effect of community participation in designing health messages before the introduction of prevention intervention for leprosy. Involving the community in structured interviews and focus group discussions for designing health messages and screening for leprosy was successful and enhanced a high rate of participation for screening and uptake of single-dose rifampicin as post-exposure prophylaxis. According to the key informants from the community, the provision of SDR-PEP generated hope for the elimination of leprosy disease and was linked with services reinforced by trained health staff. Health education considered misconceptions and beliefs expressed during structured interviews and focus group discussions that allow reinforcing counseling about the mode of transmission, the risk factors in developing the disease, that leprosy is curable, and the availability of health services, including specialized care. Community participation allowed feasibility and acceptance of SDR-PEP as all new leprosy patients detected during the study period were visited, and contacts eligible for SDR-PEP accepted it. Also, SDR-PEP was well tolerated, as only minor side effects were reported.

According to the health staff interviewed, the National Leprosy Program needs to reinforce key programmatic activities such as the provision of health supplies, training, supervision, and a referral system. These issues were solved before the active detection and post-exposure prophylaxis started by training sessions, the provision of diagnosis material, and the organizing referrals to Jorochito Dermatological Hospital and played an important role in achieving high acceptance and motivated health staff involvement.

Among six new cases detected, half had MB form and visible and permanent deformities illustrating late diagnosis. We found one new patient who was 18 years old, and this could indicate a relatively recent transmission. These two elements indicate the need to reinforce active contact tracing. There are opportunities for screening and the provision of care; for instance, a community health program that deploys health staff to assess family health by door-to-door household visits [18] should be integrated into leprosy control.

Our study had some limitations; convenience sampling for the qualitative assessment of knowledge, attitude, and practices might have introduced selection bias. However, we included a larger number of key informants and health staff for the structured interview that lately was coherent with the themes debated during the focus group discussions, even during the restrictions of the COVID-19 pandemic.

Designing the road map for the elimination of leprosy in Bolivia should include people affected by disability, as expressed by the advocacy movement “Nothing about us without us” [19] as the positive effect of involving people affected by leprosy in health programs was documented [20].

## 5. Conclusions

Community participation in designing prevention activities was successful in a pilot study including three health districts of Santa Cruz, the region most endemic of leprosy in Bolivia. The provision of SDR-PEP was feasible; it was highly accepted by the community and is well tolerated. Misbeliefs and a lack of knowledge among community members and health professionals is an important issue. Also, programmatic activities should be reinforced for the scale up of SDR-PEP. Mapping for targeting clusters might enhance the effectiveness of SDR-PEP and should be included in the roadmap for the elimination of leprosy in Bolivia.

## Figures and Tables

**Table 1:** Sociodemographic characteristics of key informants with structured interviews; Santa-Cruz, Bolivia.

Gender	N = 115	(%)
Female	68	(59)
Male	47	(41)
Age categories		
17–25	18	(16)
26–40	45	(39)
41–60	52	(45)
Residence		
Urban	43	(37)
Rural	72	(63)
Language		
Spanish	87	(76)
Spanish and Qechua	21	(18)
Spanish and others ^1^	7	(6)
Education		
Illiterate	2	(2)
Primary school	45	(39)
Secondary school	42	(37)
Technical Level	16	(14)
Master Level	10	(9)

^1^ Aimará, Guaraní.

**Table 2:** Sociodemographic characteristics of health staff with structured interviews; Santa Cruz, Bolivia.

Item	MD ^1^	(N = 142)	Nurses	(N = 75)
Gender	N°	(%)	N°	(%)
Female	45	(31.7)	68	(91)
Male	97	(68.3)	7	(9)
Age categories				
<25	16	(11.3)	8	(11)
26–35	41	(28.9)	17	(23)
36–45	49	(34.5)	23	(31)
≥46	36	(25.3)	27	(36)
Residence				
Urban	58	(40.8)	31	(41)
Rural	84	(59.2)	44	(59)
Years of work				
3	29	(20.4)	14	(19)
4–6	65	(45.8)	25	(33)
7–10	28	(19.7)	24	(32)
≥11	20	(14.1)	12	(16)

^1^ Medical doctor.

**Table 3:** Knowledge, attitude, and practices of community representatives and health staff regarding leprosy; Santa Cruz, Bolivia.

Key Informants			Health Staff		
**Knowledge**	**N 115**			**N 217**	
**Source:**	**N°**	**(%)**		**N°**	**(%)**
Family or neighbor with leprosy	20	(17)	Undergraduate training	54	(25)
Friends	52	(45)	Working experience	111	(51)
Social media	43	(38)	Reviewing literature, health brochures, etc.	52	(24)
Clinical manifestations:					
Skin, neural signs, and mutilation	48	(42)		217	(100)
Does not know	67	(58)			
Etiology:					
Non-biological ^1^	68	(59)	*Mycobacterium leprae*	217	(100)
Modifiable risk factors ^2^	30	(26)			
Atmospheric and other telluric conditions	17	(15)			
**Attitudes ^3^**	**N 436**			**N 434**	
Fear of leprosy: ^4^					
1 (Strongly disagree)	6	(1)	1 (Strongly disagree)	304	(70)
2 (Disagree)	14	(3)	2 (Disagree)	86	(20)
3 (Neutral)	81	(19)	3 (Neutral)	22	(5)
4 (Agree)	160	(37)	4 (Agree)	14	(3)
5 (Strongly agree)	175	(40)	5 (Strongly agree)	8	(2)
**Practices ^3^**	**N 385**			**N 512**	
Isolate and hide leprosy			Capacity for management		
1 (Strongly disagree)	9	(2)	1 (Strongly disagree)	12	(2)
2 (Disagree)	40	(10)	2 (Disagree)	22	(4)
3 (Neutral)	78	(20)	3 (Neutral)	70	(14)
4 (Agree)	168	(44)	4 (Agree)	248	(48)
5 (Strongly agree)	90	(23)	5 (Strongly agree)	160	(31)
**Access to care ^3,5^**	**N 443**		**Programmatic access ^7^**	**N 1181**	
1 (Strongly disagree)	71	(16)	1 (Strongly disagree)	63	(5)
2 (Disagree)	256	(58)	2 (Disagree)	186	(16)
3 (Neutral)	45	(10)	3 (Neutral)	540	(46)
4 (Agree)	36	(8)	4 (Agree)	232	(20)
5 (Strongly agree)	35	(8)	5 (Strongly agree)	160	(14)
**Prevention ^3,6^**	**N 863**		**Prevention ^8^**	**N 604**	
1 (Strongly disagree)	5	(1)	1 (Strongly disagree)	98	(16)
2 (Disagree)	24	(3)	2 (Disagree)	290	(48)
3 (Neutral)	141	(16)	3 (Neutral)	111	(18)
4 (Agree)	328	(38)	4 (Agree)	80	(13)
5 (Strongly agree)	365	(42)	5 (Strongly agree)	25	(4)

^1^ Superstition, witchcraft, and spells. ^2^ Malnutrition, alcoholism. ^3^ According to Likert scale. ^4^ Fear because severe, deadly, and highly contagious. ^5^ Including health promotion, education, and specific care by government and non-governmental organizations. ^6^ Including post-exposure prophylaxis and qualified health staff. ^7^ Includes equipment, supplies for diagnosis and wound care, training by the leprosy program, and feedback from the referral system. ^8^ Includes health education material and supervision.

**Table 4:** Sociodemographic characteristics of key informants who participated in focus group discussion.

Gender	N = 65	(%)
Female	39	(60)
Male	26	(40)
Age categories		
17–25	14	(22)
26–40	33	(51)
41–60	18	(28)
Residence		
Urban	28	(43)
Rural	37	(57)
Language		
Spanish	43	(66)
Spanish and Qechua	16	(25)
Spanish and others ^1^	6	(9)
Education		
Illiterate	2	(3)
Primary school	42	(65)
Secondary school	14	(22)
Technical Level	4	(6)
Master Level	3	(5)
Occupation		
Housework	16	(25)
Agriculture	19	(29)
Informal trade	15	(23)
Artisan	11	(17)
Professional ^2^	4	(6)

^1^ Aimará, Guaraní, etc. ^2^ School teachers, engineers.

## Data Availability

The database supporting the findings of this publication is stored at the Damien Foundation, Belgium, and will not be made openly accessible because of ethical and privacy concerns. Nevertheless, data can be made available after approval of a motivated and written request to MTU@damiaanactie.be.
